# Sternoclavicular Joint Tubercular Abscess in a Patient with Spondyloarthritis on Tofacitinib: A Case Report

**DOI:** 10.31138/mjr.270823.sjt

**Published:** 2023-08-27

**Authors:** B Sai Sunil, Prasanta Padhan, Debashis Maikap

**Affiliations:** Department of Clinical Immunology and Rheumatology, Kalinga Institute of Medical Sciences, KIIT University, Bhubaneswar, Odisha, India

**Keywords:** spondyloarthritis, tuberculosis, sternoclavicular joint

## PRESENTATION

**Figure 1.**
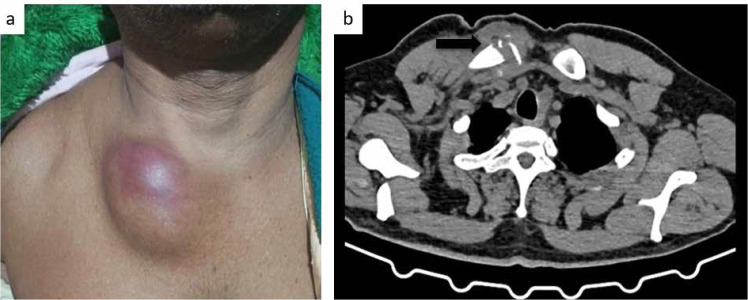
**(A)** Clinical picture showing erythematous swelling over right sternoclavicular joint region. **(B)** CT scan of the chest showing lytic lesions with adjacent few tiny bone fragments and oedematous soft tissue planes noted at the proximal end of the right clavicle.

A 52-year-old male with axial spondyloarthritis presented to us with a painless swelling in the right sternoclavicular joint for one month. Over the past week, the patient reported an increase in the size of the swelling. He had no other symptoms such as fever or weight loss. The patient was taking tofacitinib for the last six months after ruling out latent tuberculosis with a negative Mantoux test and normal chest X-ray. Local examination revealed a 3x4 cm swelling in the region of the right sternoclavicular joint that was erythematous and tender **(Figure 1A)**. The systemic clinical examination and laboratory workup were unremarkable except for raised C-reactive protein (CRP) levels of 32 mg/L (normal < 6 mg/L) and an Erythrocyte sedimentation rate (ESR) of 50 mm in the first hour. The interferon-gamma (IFN-γ) release assay (IGRA), test was positive. A chest radiograph (PA view) revealed normal lung parenchyma. A computerised tomography scan of the chest revealed lytic lesions with adjacent few tiny bone fragments and oedematous soft tissue planes were noted in the proximal end of the right clavicle **(Figure 1B)**. An incision and drainage procedure was performed on the affected site. Gram stain and culture of the pus were negative for bacteria. However, the Ziehl-Neelsen stain was positive for acid-fast bacilli, and the blood culture showed no evidence of bacterial growth. Furthermore, the tuberculosis (TB) polymerase chain reaction (PCR) test confirmed the presence of Mycobacterium tuberculosis. The patient was registered under the national tuberculosis elimination programme (NTEP) and started on treatment with Anti-tubercular therapy. On follow-up at 6 months, the wound had healed. The patient received a full course of anti-tubercular therapy (ATT) for 12 months, comprising two months of HRZE followed by ten months of HRE.

## DISCUSSION

Tuberculosis affecting bones and joints is rare, constituting only 1% to 3% of all tuberculosis cases.^1^ Sternum and sternoclavicular regions are affected in approximately 1% of musculoskeletal tuberculosis cases.^1,2^ Patients with sternoclavicular joint tuberculosis may experience painful swelling, painless swelling, or sinus, although the latter is rare.^3^

Janus kinase (JAK) inhibitors have recently been introduced in the management of spondyloarthritis, but their use increases the risk of TB infections. Tofacitinib is a JAK inhibitor, which preferentially inhibits JAK3 and JAK1, modulating the immune response via downregulation of several cytokines (eg, interleukins [ILs] 2, 4, 7, 9, 15 and 21) that are integral to lymphocyte development and function. It indirectly affects TNF by blocking interferons. It has been hypothesised that blockade of IL-12 or IL-23 (which act through JAK2/TYK2) might lead to inhibition of IFNγ production by T cells. TNFα is essential in granuloma formation while IFNγ is vital in preventing TB dissemination.^4^

Before starting JAK inhibitors, it is important to screen for TB infection using both Interferon-gamma release assay (IGRA)/Quantiferon TB gold, and Mantoux tests as per an Egyptian study.^5^ Relying solely on the Mantoux test may miss cases of latent tuberculosis in TB-endemic areas like India.

A thorough evaluation of the potential risks and benefits, along with regular monitoring for TB infection, is crucial to ensure the safe and effective use of JAK inhibitors in clinical practice.

## References

[1] Watts HG Robert L. Tuberculosis of Bones and Joints. J Bone Joint Surg Am 1996 Feb;78(2):288–98. 8609123 10.2106/00004623-199602000-00019

[2] Dhillon MS Gupta RK Bahadur R Nagi ON. Tuberculosis of the sterno-clavicular joints. Acta Orthop Scand 2001;72:514–7. 11728080 10.1080/000164701753532862

[3] Shah J Patkar D Parikh B Parmar H Varma R Patankar T Tuberculosis of the sternum and clavicle: Imaging findings in 15 patients. Skeletal Radiol 2000;29:447–53. 11026712 10.1007/s002560000207

[4] Schwartz DM Bonelli M Gadina M O’Shea JJ. Type I/II cytokines, JAKs, and new strategies for treating autoimmune diseases. Nat Rev Rheumatol 2016;12:25–36. 26633291 10.1038/nrrheum.2015.167 PMC4688091

[5] Sellami M Fazaa A Cheikh M Miladi S Ouenniche K Ennaifer R Screening for latent tuberculosis infection prior to biologic therapy in patients with chronic immune-mediated inflammatory diseases (IMID): Interferon-gamma release assay (IGRA) versus tuberculin skin test (TST). Egyptian Rheumatol 2019 Jul 1;41(3):225–30.

